# A novel approach to full-mouth rehabilitation of dentinogenesis imperfecta type II: Case series with review of literature

**DOI:** 10.1097/MD.0000000000036882

**Published:** 2024-01-26

**Authors:** Yizhou Zhang, Xiaoting Jin, Zhengyi Zhang, Sai Hu, Wenxiang Jiang, Haisong Pan, Ling Zhang, Baiping Fu

**Affiliations:** a Stomatology Hospital, School of Stomatology, Zhejiang University School of Medicine, Zhejiang Provincial Clinical Research Center for Oral Diseases, Key Laboratory of Oral Biomedical Research of Zhejiang Province, Cancer Center of Zhejiang University, Engineering Research Center of Oral Biomaterials and Devices of Zhejiang Province, Hangzhou, China; b The Fourth Affiliated Hospital, Zhejiang University School of Medicine, Yiwu, China; c 6D Dental Tech CO., Ltd, Hangzhou, China.

**Keywords:** CAD/CAM template, dentinogenesis imperfecta, obliteration, review, tooth wear

## Abstract

**Rationale:**

Dentinogenesis imperfecta (DI) is an autosomal-dominant disorder. The most common clinical manifestations, including obliterated tooth tissues and severe tooth wear, usually lead to tooth extractions. It remains a great challenge for dentists to preserve the residual tooth tissue and establish the esthetics and occlusion of dentitions.

**Patients concerns:**

25-year-old twin sisters, who had suffered from dentinogenesis imperfecta type II for more than 10 years, presented with continuous tooth wear and discomfort from wearing a removable partial denture for more than 3 years.

**Diagnosis:**

Intraoral examination showed extensive tooth wear with enamel exfoliation and typical amber-brown color with an opalescent discoloration. Their panoramic radiographs revealed completely obliterated tooth tissues and severe tooth wear.

**Interventions and outcomes:**

The dentitions were restored with post-and-core crowns and pin lays after preparing root post paths and pin holes guided by computer-aided design/computer-aided manufacturing (CAD/CAM) procedures, resulting in a successful repair.

**Lessons:**

Severe tooth wear and tooth tissue obliteration are typical clinical manifestations in DI-affected dentitions, increasing the complexity and difficulty in dental restorations. Early diagnosis and appropriate treatments are essential to achieve a favorable prognosis. CAD/CAM procedures, permitting accurate and effective treatment, possess promising potential in the treatment of DI-affected dentitions.

## 1. Introduction

Dentinogenesis imperfecta (DI) is a localized mesodermal dysplasia that is characterized by dentin hypomineralization and abnormal dentin structure.^[1,2]^ Besides, it is inherited in an autosomal dominant pattern with high penetrance and low mutation rate.^[3]^ The incidence of DI has been reported to be between 1 in 6000 and 1 in 8000, and DI type II was reported to have a prevalence of 0.0022% in a Swedish study.^[4,5]^ DI affects both primary and permanent teeth, but even though primary teeth are often more severely affected.^[5–7]^ Shields classification system categorizes DI into 3 types.^[8]^ DI type I is associated with osteogenesis imperfecta.^[4]^ DI type II possesses many clinical and radiographic features similar to DI type I without osteogenesis imperfecta.^[4,9]^ Type-III, a phenotype found in a tri-racial population in Maryland and Washington DC, is characterized by large pulp chambers.^[4]^ Clinically, the affected teeth are discolored, ranging from opalescent blue-gray to dark yellow-brown.^[10]^ The enamel is prone to chip away from dentin and the exposed dentin is susceptible to rapid tooth wear which leads to a decrease of vertical dimension.^[7,11–13]^ Structural defects are characterized by bulbous crowns, cervical constrictions, small pulp chambers, and partially or completely obliterated root canals.^[7]^

Early diagnosis and appropriate treatments are essential to achieve a favorable prognosis. The treatment of DI is often complicated and case-dependent. Prompt interventions are essential to protect primary and mixed dentitions with metallic restorations. Generally, the treatment aims to preserve the tooth tissues, prevent further loss of the vertical dimension of occlusion, and restore tooth functions and esthetics.^[14]^ But when the severe tooth wear has occurred in permanent dentition, the rehabilitation could be complex and unpredictable.^[15]^ This scenario requires other restorative methods to achieve a predictable rehabilitation.

Recent advances in computer-aided design/computer-aided manufacturing (CAD/CAM) technology have generated a revolution in modern dentistry, enabling more accurate treatment and surgery. CAD/CAM templates have been applied in various fields, such as implant surgery, guided endodontics, orthognathic surgery and so on.^[15]^ The templates permit the personalized design of dental appliances, simplify the implementation of complex procedures with less error, and overcome the limitations in freehand operation.^[15]^

The purpose of our clinical reports was to present tooth restorations in 2 cases of twin sisters diagnosed with DI type II, with the aid of CAD/CAM templates (Soft tissue - Supported Guide, 6D-Dental Medical Technology Co., Ltd, Hangzhou, China) to permit the accurate preparation of root post paths and pin holes in obliterated tooth tissues, facilitating the subsequent restoration of metal-ceramic post-and-core crowns and pin lays. A review of the state-of-the-art of the treatments in DI-affected dentitions during different periods with typical clinical manifestations was performed.

## 2. Case presentation

**Figure 1.**
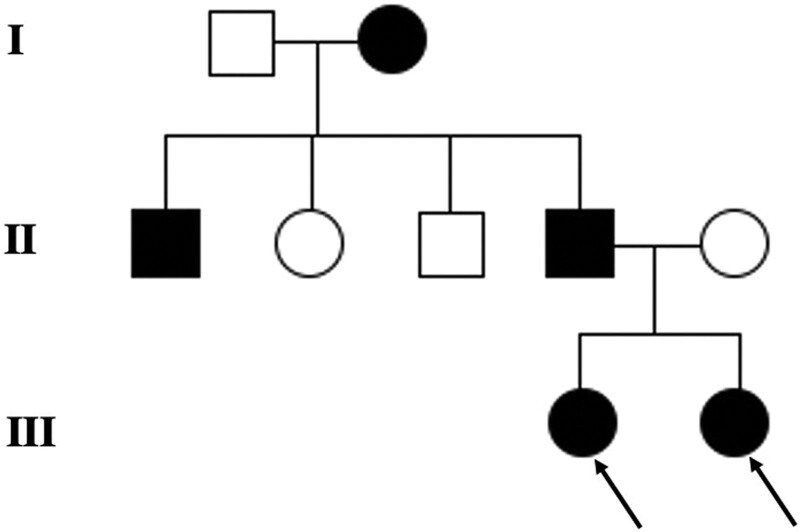
Pedigree of the patients family with arrows referring to the patients. Darkened = affected; clear = unaffected, squares = males; circles = females. I = 1st generation; II = 2nd generation; III = 3rd generation.

**Figure 2.**
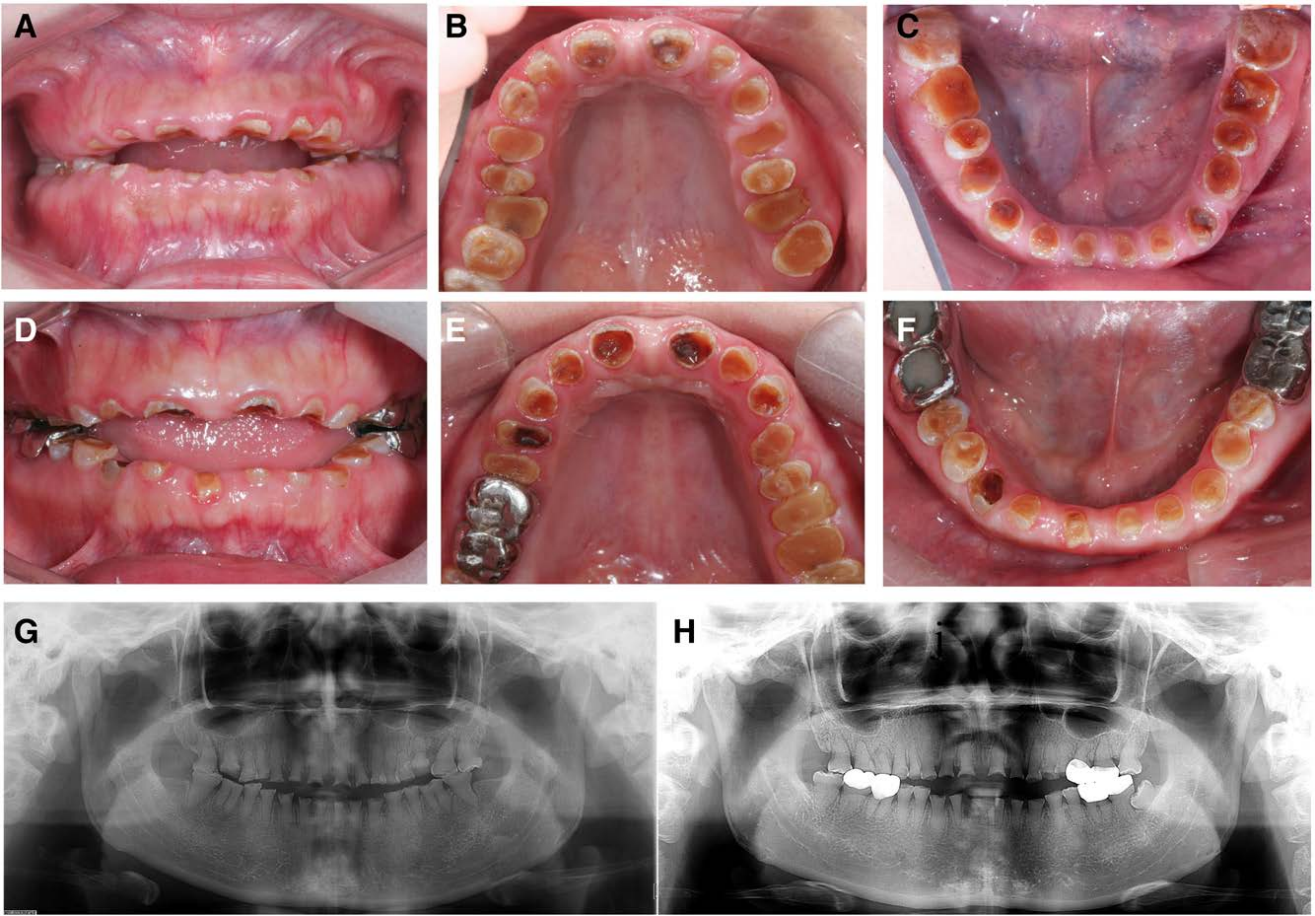
Pretreatment intraoral views and panoramic radiographs of the younger sister (panels A–C, G) and the elder one (panels D–F, H). The teeth showed severe tooth wear and dental caries indicating anterior open bite (panels A–F). For the elder sister, the teeth #16, 17, 36, and 37 were restored with full metal crowns, and the teeth # 46 and 47 were restored with acrylic resin veneer metal crowns (panels E, F), while preserving posterior occlusal contact relationships and VDO (panel D). Radiographs showed that all teeth have completely obliterated pulp chambers and root canals (panels G, H). VDO = vertical dimension of occlusion.

The 25-year-old twin sisters, with the diagnosis of DI type II for more than 10 years, visited the Stomatology Hospital, School of Stomatology, Zhejiang University School of Medicine. They mainly complained about the consecutive tooth wear, and uncomfortableness of removable partial dentures that had been worn for more than 3 years. They both have not reported any other systemic diseases. The family medical history revealed that their father, paternal grandmother and one of their father’s siblings had similar dental symptoms (Fig. 1). Extraoral examination revealed that the patients had no perceptible loss of occlusal vertical dimension without pain and clicking of temporomandibular joint. Intraoral examination showed extensive tooth wear with enamel exfoliation and typical amber-brown color with an opalescent discoloration (Fig. 2A–F). Their panoramic radiographs revealed complete obliterated tooth tissues (Fig. 2G, H). Periapical lesions on the tooth #16 were detected in the elder sister (Fig. 2H). The patient involved in the study have signed a written informed consent that the health-related data would be used for research purposes and photographs would be published in this way. This study was approved by the Institutional Ethic Board of the Affiliated Stomatology Hospital, Zhejiang University School of Medicine, Hangzhou, China (No. 2020-16).

### 2.1. Treatment plan

The patient consent form was taken. Restorative options, including implant-supported restoration, and post-and-core crowns and pin lays restoration, were proposed. The latter was accepted. We reminded them that a deep bite might occur in anterior region, an orthodontic treatment was better to be performed, but they refused because of its long therapeutic time and high costs. According to the treatment plans, routine oral hygiene instructions were given and ultrasonic scaling was performed firstly. CAD/CAM templates were planned to guide the preparation of root post paths and pin holes in obliterated tooth tissues. The posterior teeth were restored with pin lays, and anterior teeth were restored with post-and-core 2-unit crowns. All-ceramic restorations were recommended but metal-ceramic ones were finally chosen because of their limited financial resources. For the elder sister, teeth #31 and 41 were replaced with 2 implants because the roots were too short. The tooth #16 with periapical lesions was left untreated since endodontic treatment was difficult to conduct due to the complete obliteration of tooth tissues, and the extraction may be performed if the lesions progressed.

### 2.2. Building the CAD/CAM template

**Figure 3.**
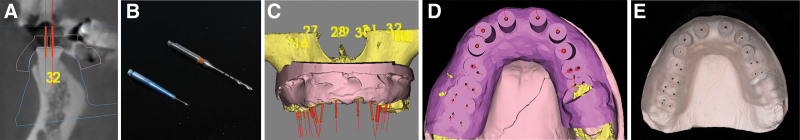
The brief procedures of an upper CAD/CAM template construction. (A) Two pin holes were designed in a lower premolar based on the CBCT data. (B) The drills for preparation (left: for pin holes, 0.6 mm in diameter; right: for root post paths, 0.9 mm in diameter). (C) Guiding sleeves were deigned after the CBCT data and digital impression data were matched based on radiopaque structures. (D) The digital template was finally designed. (E) The template was printed with the designed sleeves incorporated. CAD/CAM = computer-aided design/computer-aided manufacturing, CBCT = cone-beam computed tomography.

A high-resolution cone-beam computed tomography (CBCT) scan was taken using the NewTom VGi (NewTom, Verona, Italy), operating at 110 kVp, 1.64 mA, 3.6 seconds with a FOV of 15 × 12 cm and voxel size 0.3 mm. The design of the root post paths and pin holes was determined according to the diameter and length of each root obtained from the CBCT data (Fig. 3A). Post paths for anterior teeth were designed with a diameter of 0.9 mm in the middle of the root leaving 1 mm of root wall thickness and 3 mm to the apical point. Pin holes were designed with 2 pins for premolars leaving 2 mm from the buccal or lingual side in middle line from the mesiodistal view, and 3 or 4 pins for the molars leaving 2 mm from the axial walls. Pin holes should be parallel to each other in the same tooth with a diameter of 0.6 mm and a depth of 2 mm. The post drill (ParaPost, Ref.: P423; Coltene/Whaledent AG, Altstätten, Switzerland) for root post path preparation and the pin drill (Self-threading screw, Ref.: K11-60; Westlake Biological Materials Co., Ltd, Hangzhou, China) for pin hole preparation were used in this study (Fig. 3B). After impressions of the upper and lower dentitions were taken using silicone rubber impression materials (3M ESPE Express, St. Paul, MN), stone casts were obtained, and scanned using an optical scanner (D700, 3Shape, Copenhagen, Denmark) with an accuracy of 20 µm. Image datasets including CBCT data and digital impression data were uploaded to design software (Version2.0, 6D-Dental Medical Technology Co., Ltd, Hangzhou, China), and their images matched based on radiopaque dental structures. The superimposition of the drills was positioned in the middle of the designed roots (Fig. 3C). Guiding sleeves (0.9 mm internal diameter, 1.7 mm external diameter for post paths; 0.6 mm internal diameter, 1.4 mm external diameter for pin holes) were customized for each drill, and templates were designed (Fig. 3D). Data was exported as STL files and printed using a 3D printer (Objet 350 Connex3, Stratasys), and the designed sleeves were incorporated into the templates (Fig. 3E).

### 2.3. Prosthodontic treatment

**Figure 4.**
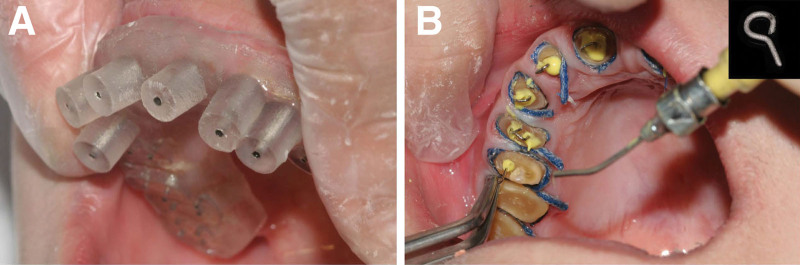
(A) The drills used (left: for pin holes preparation, 0.6 mm in diameter; right: for post paths preparation, 0.9 mm in diameter). (B) The maxillary CAD/CAM template. (C) Positioning of the template on the dentition. (D) Impression making: the impression auxiliary pin was insert into pin holes (shown in the upper right-hand corner) while injecting the light body over pin holes. CAD/CAM = computer-aided design/computer-aided manufacturing.

The templates were positioned on the dentition and fitness was checked (Fig. 4A). The drills were used to prepare root post paths and pin holes until the end of the drill shafts touched the sleeves. One-step impression technique was used with silicone rubber impression materials for anterior post paths with dentition, and after that, a stone cast was obtained. Upper and lower casts were mounted on an articulator according to the occlusal relationship record. Post-and-cores of anterior teeth were fabricated, tried in and cemented with glass ionomer cement (GIC) (GC Fuji I, Japan). The finishing margin was prepared with a circumferential chamfer of approximately 0.5 mm width at 0.5 mm of subgingival. An orthodontic round wire (0.020’’, F810-35, Westlake Biological Materials Co., Ltd, Hangzhou, China) was bent into approximately 3 mm pin with a small loop, which served as impression auxiliary pins, and were sandblasted before use. Gingival retraction with the double cord technique (#000 and #1, Ultrapak, Ultradent Products, Inc., UT) was used in this study. Auxiliary pins were inserted into pin holes immediately after the light body was injected over pin holes and directly into post paths using a modified No. 9 needle (Fig. 4B). One-step impression technique was used for both upper and lower dentitions, and their casts were obtained. Routine occlusal relationships were recorded and metal-ceramic pin lays and crowns were fabricated, tried in, marginal adaption checked in place, and occlusal adjustment was performed. The 2-unit crowns of anterior teeth were finally cemented with the GIC.

The full-mouth rehabilitation for the younger sister was finished as above mentioned. But the treatment for the elder sister was more complex. Two dental implants (SLA, bone level, Ref.: 021.2514, Institution Straumann AG, Basel, Switzerland) were inserted in the site of teeth #31 and 41 after bone grafting, which used artificial bone powders and collagen membranes (bone powder: Bio-Oss, Geistlich, Switzerland; collagen membrane: Bio-Gide, Geistlich, Switzerland). Two models of each jaw were made and a check bite was performed. The models were mounted in an articulator and scanned using an optical scanner. Afterwards, maxillary anterior transitional crowns and a mandibular implant-supported transitional cantilever bridge were designed and fabricated with CAD/CAM technologies. The transitional dentures and definitive restorations on premolars in both jaws were subsequently restored and 3 months later, transitional dentures were replaced with definitive ones.

### 2.4. Clinical outcomes

**Figure 5.**
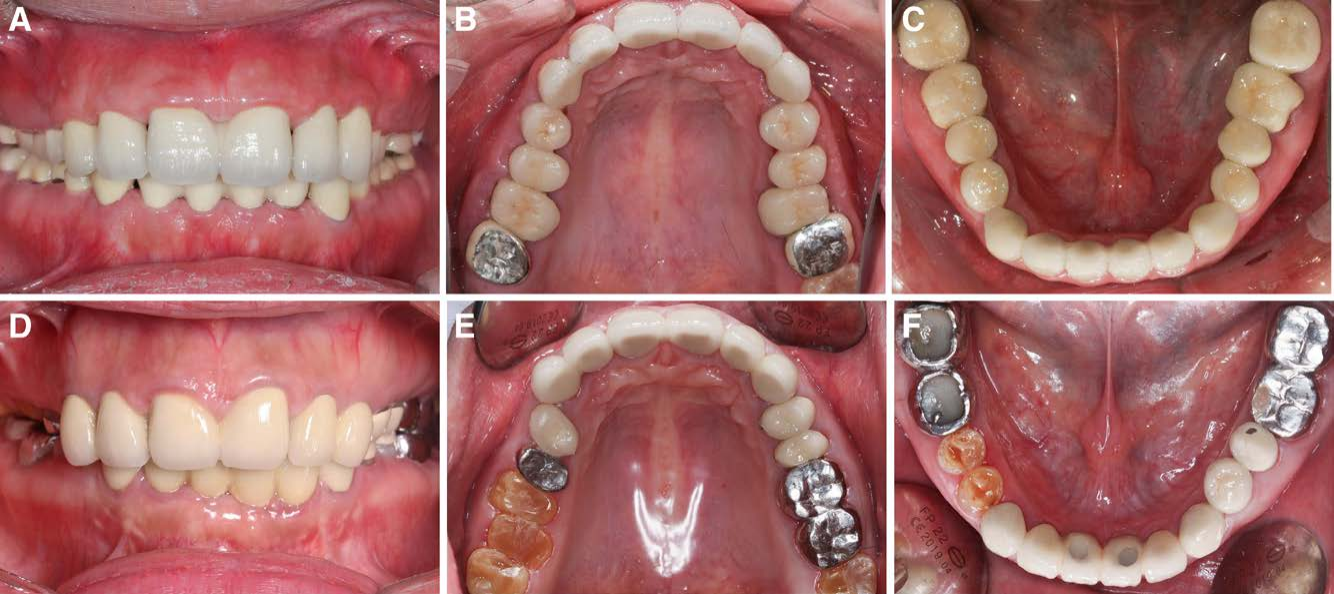
Intraoral views after definitive restoration. (A–C) The younger sister. (D–F) The elder sister.

Posttreatment photographs showed that the definitive restorations were satisfactory, and the patients were satisfied with the treatment results (Fig. 5). The patients were recalled at 3, 6 and 12 months in the first year, and once a year afterwards. For the younger sister, the 2-unit crowns of the teeth #11 and 21 were dislodged at 37 months. The dislodged crowns were re-cemented. The pin lay of the tooth #14 was broken at 40 months due to the direct chewing of hickory. The pin lay was refabricated and cemented. For the elder sister, the post-and-core crowns of the teeth #11 and 21 were dislodged at 20 months. The 2-unit crowns of the teeth #12 and 13, 22 and 23 were dislodged at 23 months. The fixed prothesis were cleaned and re-cemented.

## 3. Literature search

A literature search was conducted on the treatment of DI-affected dentitions with typical clinical manifestations, especially tooth wear and/or tooth tissue obliteration. The electronic database Web of Science were searched from inception to 31st January 2023 using the following MeSH terms: “dentinogenesis imperfecta”, “treatment”, and “therapy”. It was supplemented by manual searches in the reference lists of the relevant articles. The search retrieved 300 results. Different types of peer-reviewed publications, only in the English language, were screened as follows: indexed reviews, case reports and case series. After screening the title, abstract and full-text, 92 relevant full-length articles were included. Among them, 28 cases that reported the treatment of DI-affected dentitions in different periods were selected, summarized and listed in Tables 1–3.

## 4. Discussion

DI is a genetic disorder that affects the structural integrity of the dentin.^[10]^ The most common features of DI include tooth wear and obliterated tooth tissues, which account for the increasing complexity and unpredictability of the restorations in DI-affected dentitions. In our clinical reports, the patients were examined with typical clinical manifestations of DI type II, tooth extractions and implant-supported fixed or removable prostheses were proposed as an alternative. But full-mouth rehabilitation was finally achieved assisted with CAD/CAM procedures, preserving the residual tooth tissues with acceptable esthetics and functions. Our reports add new elements to the literature regarding the dental management of DI patients. In order to identify the treatments in different periods and conditions of DI, a literature review was required.

Early identification and preventive interventions are critical for DI children to intercept tooth wear and prevent pulp damage.^[3,18,22]^ Otherwise, the unrestorable deterioration of dentitions may affect the normal growth of facial bones and temporomandibular joint, leading to an unesthetic appearance and causing psychological problems.^[15]^ Maintenance of good oral hygiene, pit and fissure sealing and topical fluoride application as effective early preventive approaches are strongly recommended.^[22]^ Early restoration of primary molars and permanent first molars during mixed dentition with stainless steel crowns is an effective method to prevent tooth wear.^[3,23,24]^ When the children reach adulthood, stainless steel crowns can be replaced with full crowns.^[25]^ Primary anterior teeth are restored with aesthetic preformed pediatric crowns such as resin composite strip crowns, polycarbonate crowns and zirconia crowns.^[26,27]^ However, insufficient evidence support any method or particular material for the primary anterior teeth.^[28]^ Permanent anterior teeth should be restored with composite as soon as eruption starts to reduce tooth wear, and can also be protected with celluloid strip crowns or polycarbonate crowns, and porcelain crowns at a later stage.^[24]^ Teeth that cannot be restored with crowns can be restored with overlay dentures on the residual crowns and roots.^[29]^

Various approaches for managing DI-affected permanent dentitions have been documented. Routine restorative techniques including composite restorations and veneers might be used for the dentitions without enamel fracture or extensive wear.^[30,31]^ The full-coverage crowns are the preferred choice for the teeth with extensive tooth wear.^[30,32]^ However, the worn teeth are always unable to provide sufficient retention for crowns restoration. Soliman S et al^[13]^ used self-threading parapulpal retentive pins in combination with composite resin buildup cores, though they do not possess stronger retention than conventional posts and cores. Crown lengthening procedures on teeth without bulbous shape or short roots to obtain sufficient clinical crown height is an another approach to obtain sufficient retention for crown restorations.^[14,31,33,34]^ Splinted crowns are sometimes recommended to reduce the risk of individual root fracture.^[35]^ With the development of technology, indirect adhesive restorations can replace crowns in minimal invasive preparations on DI-affected teeth. Indirect composite resin overlays have been used for DI patients without adhesive failure for 13 years.^[31]^ When the tooth loss occur, the dentitions could be restored with fixed partial dentures and implant-supported crowns or bridges.^[32,33,36,37]^ However, fixed partial dentures might exert extreme stress on the teeth and increase the risks of root fractures.^[36]^ The implant-supported fixed or removable dentures is a predictable approach, but the cost is expensive and the implant placement should be prudently planned.^[14]^ Overdenture is a common removable denture with the underlying residual tooth tissues restored with copings.^[36]^ It is a comparatively cheaper prosthesis, however, it might hamper oral hygiene.^[7,14,38]^

The most recent cases reports focus on the application of digital workflows and multidisciplinary treatments in DI-affected dentitions. Fan F et al^[34]^ used digital smile design to design the esthetics of the anterior teeth, which were severely worn with unesthetic appearance. A multidisciplinary treatment protocol focusing on prosthodontics proved highly effective in the esthetic restorations. Shi S et al^[30]^ used digital techniques including digital smile design, the ARCUSdigma axiograph and CAD/CAM to achieve accurate occlusion and improve the efficacy of esthetic rehabilitation in DI. CAD/CAM technology has been reported to assist in the fabrication of monolithic prostheses with reduced thickness for a moderately cooperative DI child with severe tooth wear.^[20]^ Another case reported that a minimally invasive and effective rehabilitation in a DI child was achieved through digital workflows, including intraoral scanning and individual crowns manufacturing via CAD/CAM.^[30]^ Azpiazu-Flores FX et al presented the interdisciplinary management of a DI patient. CAD-CAM procedures were applied to fabricate devices that aided planning, assisted intermaxillary fixation and implant placement, and enabled the accurate establishment of the definitive full-arch prostheses.

In our clinical reports, the 2 patients were examined with severely affected dentitions, requiring complex reconstructions involving advanced prosthetic treatments. The CAD/CAM procedures made it feasible. In our cases, root post paths and pin holes were accurately prepared in obliterated pulp chambers and root canals assisted with the CAD/CAM templates. Other than high accuracy, minimally invasive preparation, reduced chair time and decreased technique sensitivity were also achieved. However, without the templates, the preparation could be difficult and unpredictable, and root perforation may occur, which could lead to tooth extraction. This is because the roots are thin and the preparation direction of paths and holes is difficult to control using the free hand. With the subsequent restoration of metal-ceramic post-and-core crowns and pin lays, maximizing residual tooth hard tissues preservation and full-mouth rehabilitation were both achieved. Generally. CAD/CAM templates have been widely used in dentistry, but they have not been reported to guide the accurate root post paths and pin holes preparation in fully obliterated tooth tissues in DI patients.

The treatment plans including tooth extractions with implants replacement were proposed at first. But the patients urged to preserve their natural teeth even for a short time, and the high costs of implant restorations were unaffordable to them. Therefore, they chose the traditional post-and-core crowns and pin lays restorations. The root lengths of anterior teeth were basically sufficient for post-and-core restorations but insufficient for crown lengthening procedures, therefore, it was not performed. Two-unit crowns were used in anterior region to reduce the risk of individual root fracture, because of the occurrence of a moderate deep bite. Pin lays were used in posterior teeth in order to enhance the mechanical retention force. Overlays or occlusal veneers were not used because of the insufficient adhesion between the restorations and malformed tooth tissue. Although the pins were thin and short, we didn’t find any fracture under normal conditions during the 3-year follow-up, expect a fracture in the 40^th^ month associated with chewing of hard hickory. The restorations were made in metal-ceramic rather than all-ceramic because of the high costs of the latter, although the latter could provide better esthetics.

Follow-up studies in the cases revealed several problems. Prosthesis dislodgement occurred severally in both patients during the 3-year follow-up period, which should be attributed to the severe tooth wear of their dentitions, and the decrease in bond strength between the cement and malformed hard tissue.^[13]^ The GIC rather than resin-based cement was used, because both gained similar bond performance for cast restorations in DI.^[14]^ The prothesis dislodgement mainly occurred in the anterior region, which might have resulted from occlusal interference by a moderate deep bite. Orthodontic interventions could effectively eliminate the deep bite, but were not considered because of long therapeutic time and high costs.

Clinically, clinicians always provide the best treatment plans for patients, but usually, they are not accepted by patients, limited by the costs, patients desires and clinical resources. So individual treatments were required. In our cases, some problems kept its existence in the therapeutic cycle, but our treatment seemed to be the best choice considering all the factors. Furthermore, CAD-CAM templates possess promising potential in the treatment of DI-affected dentitions.

## 5. Conclusion

DI is an autosomal dominant condition with complex manifestations that increases the difficulty and predictability of the treatment. Early diagnosis and appropriate treatments are essential to achieve a favorable prognosis. Individual treatments are required to achieve satisfactory esthetics and functions. In our cases, CAD/CAM templates guided the accurate preparation of root post paths and pin holes in obliterated tooth tissues, facilitate the full-mouth rehabilitation in 2 patients with acceptable results. We conclude that CAD/CAM technology could be recognized as a blueprint in oral rehabilitation.

**Table 1:** The summary of DI-affected primary dentitions with typical clinical manifestations and the treatments.

Author(s)-Country	Age and gender	Clinical and radiographic manifestations	Treatment	Follow-up
Sapir S et al, 2001^[3]^-Israel	An 8-mo-old girl	Tooth wear scale: 3 (the erupted teeth); wide pulp chambers and poorly calcified incisors	Stage 1: at age 18–20 mo, composite restorations (# 52–62, 72–82), preformed crowns (# 54, 64, 74, 84) Stage 2: performed crowns (# 55, 65, 75, 85); composite resin restorations (# 53, 63, 73, 83)	At 6 mo, the restorations were esthetically acceptable and functional.
Huth KC et al, 2002^[16]^-Germany	A 4-yr-old boy	Tooth wear scale: 3–4; completely obliterated pulp chambers	SSCs (# 54, 55, 64, 65, 74, 75, 84, 85); composite crowns (# 53–63, 73–83); extracted: # 73 (necrotic); intraoperative space maintainer	At 6 mo, all restorations were functional and the static and dynamic occlusion revealed no interferences.
Beltrame APC et al, 2017,^[11]^ Brazil	A 1 yr, 8-mo-old girl	Tooth wear scale: 1–3	fluoride varnish application; composite resin restorations (# 52–62); indirect composite resin restorations (# 72–82)	At 32 mo, the restorations continue to play their roles with success.
Abukabbos H et al, 2013,^[17]^ Saudi Arabia	A 4-yr-old boy	Missing teeth: # 74; Tooth wear scale: 1–2; partially obliterated pulp chmabers	SSCs restoration (#55, 54, 65, 75, 84, 85); extracted: # 64 (dentoalveolar abscess); celluloid strip crowns restoration (# 51, 52, 61, 62, 71, 72, 81, 82); composite buildup (# 53, 63, 73, 83); band-and-loop space maintainers (between teeth # 63 and 65, # 73 and 75); topical fluoride treatment	At 18 mo, good oral hygiene practice.

The severity of tooth wear was rated with the Tooth wear Index proposed by Smith BG and Knight JK in 1984^45^, estimated by the authors depending on the provided text and image information. The acronym “FPD” represents “fixed partial denture”, “P” represents “pontic”, “OP” represents “operation”, “VDO” represents “vertical dimension of occlusion”, “SSC” represents “stainless steel crown”, “GIC” represents “glass ionomer cement”.

DI = dentinogenesis imperfect, SSCs = stainless steel crowns.

**Table 2:** The summary of DI-affected mixed dentitions with typical clinical manifestations and the treatments.

Author(s)-country	Age and gender	Clinical and radiographic manifestations	Treatment	Follow-up
Delgado AC et al, 2008^[18]^-Spain	A 6-yr-old boy	Tooth wear scale: 3–4; almost complete obliterated pulp chambers and root canals	Extracted: #55, 65 (to facilitate the eruption of #16, 26); fissure sealants (# 36, 36); nickel-chromium crowns (# 74, 75, 84, 85); overdenture	Failed to follow through.
Alrashdi M et al, 2020^[19]^-Saudi Arabia	A 5-yr-old girl	Tooth wear scale: 3–4; periapical lesions: # 54–55, 65, 75; 84–85; large pulp chambers of the developing permanent buds; a loss of VDO	Extracted: # 54–55, 65, 75; 84–85; overlay denture	Not reported
Alrashdi M et al, 2020^[19]^-Saudi Arabia	A 7-yr-old boy	Tooth wear scale: 3–4; extensive caries: #16, 26, 36, 46	SSCs reatoration: # 75, 85, 16, 26, 36, 46; GIC: # 12–22, 32–42	Not reported
Millet C et al, 2020^[20]^-France	A 7-yr-old girl	Tooth wear scale: 4; no obliteration of permanent teeth pulp chambers; a slight loss of VDO	CAD/CAM complete overdentures	At 6 mo, showed good stability and no signs of complications.
Sarapultseva M et al, 2020^[21]^-Russia	A 6-yr-old boy	Tooth wear scale: 3; obliterated pulp chambers; a loss of VDO	Steel milled crowns: # 16, 55, 65, 26, 36, 46; composite resin crowns: 12, 22, 11, 21, 32, 31, 41, 42	At 3 yr, the treatment outcome was stable.

CAD/CAM = computer-aided design/computer-aided manufacturing, DI = dentinogenesis imperfect, SSCs = stainless steel crowns, VDO = vertical dimension of occlusion.

**Table 3:** The summary of DI-affected permanent dentitions with typical clinical manifestations and the treatments.

Author(s)-country	Age and gender	Clinical and radiographic manifestations	Treatment	Follow-up
Licht WS et al, 1980^[36]^-USA	A 15-yr-old girl	Tooth wear scale 3–4; pulp canals were completely obliterated except the newly erupted second molars	Extracted: #16, 46 (caries), 15 (impacted tooth), 12, 22, 32–42 (severe tooth wear); complete cast gold crowns FPD: #17P^16^P^15^14; (#17P^16^P^15^14) single crowns: #24,15,16,27; complete acrylic resin veneer gold FPD (#13P^12^11 21P^22^23); cast gold copings (#33–37, 43–45, 47) and a lower cast chrome cobalt overdenture	Not reported
Mendel RW et al, 1981^[39]^-USA	An 18-yr-old woman	Complete absence of the pulp or residual hair-like canals (all fully formed teeth); periapical cyst (#21), poor retrograde fillings (#11, 21); Steel basket crowns (#13–23), full steel crowns (#33–43, 16, 26, 36, 46)	Retrograde fillings (#11, 21), OP: periapical cyst enucleation (#21); metal crowns removal (#33–43); a supragingival acrylic splint (#33–43); extracted #36, 37, 47, 21 (loss of coronal tooth structure); crown and FPD restorations (not accurate report)	At 2 yr, a lower splint was fractured between #41 and 42, all teeth were extracted and immediate dentures were worn for 18 mo; At 4 yr, new dentures were made, the patient adapted well.
Eerikäinen E , 1981^[35]^-Finland	A 13-yr-old boy	Tooth wear scale 3–4: incisors and first molars; scale 2–3: the other teeth; obliterated pulp chambers and root canals (all the teeth)	Self-threading parapulpal retentive pins with amalgam core (#16, 26, 36, 46, and six premolars); Self-threading pins with composite resin core (#12–23, 33, 43); full gold and veneer crowns (#16–14, 24–26, 33–36, 43–46); acrylic crowns (#13-23, 32-42)	During a period of about 4 yr, no clinical failure occurred.
Henke DA et al, 1999^[33]^-USA	A 27-yr-old man	Tooth wear scale 3–4, obliterated pulp chambers and root canals	Extracted all mandibular teeth (extreme tooth wear); six implant-supported bridge (lower arch); a pin-retained and bonded amalgam core #26 (inability to perform endodontics because of obliteration); crown lengthening on all maxillary teeth; splinted crowns (#16 and 15, #11–13, #21–23, #25 and 26)	Not reported
Stephen LXG et al, 2002^[38]^-South Africa	A 16-yr-old girl	A loss of VDO; obliterated pulp chambers and root canals; a cross bite	Restored #26 (caries); Smoothed sharp ridges and reduced undercuts of the remaining upper teeth and a maxillary overdenture was delivered (to correct VDO and cross bite); porcelain-gold VMK full crowns (lower teeth)	At 6 mo, the occlusion and oral hygiene status were improved. Overdenture and crowns have been functional and comfortable for several yr (not accurate reported).
Moundouri-Andritsakis H et al, 2002^[40]^-Greece	A 13-yr-old boy	Tooth wear scale 2–3; regular pulp chambers	Orthodontic treatment (2 yr); composite restorations (2 yr); all-ceramic crowns (17–27, 37–47)	At 3 yr, no functional problems
Prabhu N et al, 2007^[41]^-Australia	A 32-yr-old man	Crowns (#26, 27); FPD (#48P^47^P^46^P^45^ 44 43 42 41); all the remaining teeth were badly broken down because of severe caries	Extracted the badly broken teeth; implant-supported overdentures for both arches	12 mo after implant placement, one upper implant was lost but didn’t affect the treatment outcome; a 9-yr recall visit showed a successful result.
Groten M, 2009^[32]^-Germany	A 14-yr-old boy	Tooth wear scale 3–4; narrow pulp chambers	Endodontic treatment on several teeth (pulp necrosis); all-ceramic zirconia posts after root canal filling; intraoral buildups (teeth with clinical height lost); crown lengthening (#32–42); single crowns (#17–27); splinted crowns (#31–34, #41–44); four-unit cantilevered FPDs (#35–37, #45–47, with an additional pontic closing the space between #34 and 35, #44 and 45)	At 6 wk, 3 minor chip fractures occurred, but it is functionally and esthetically unimportant.
Millet C et al, 2010^[42]^-France	A 9-yr-old girl	Pulp chambers were obliterated without tooth wear; #12, 22 were congenitally missing	Third molar tooth germs removal; metal-ceramic crowns (#17, 15–13, 11, 21, 23–25, 35–45)	At 10 years, crown restorations were successful.
Roh WJ et al, 2010^[43]^-South Korea	A 17-yr-old girl	Tooth wear scale: 0–2; pulpal obliteration; Class II Division 1 malocclusion; tooth fracture (#11, 21); impacted tooth (#37, 18, 28, 38, 48);	Extracted #11, 21 (tooth fracture), 25 (palatally blocked tooth), 46 (extensive caries), 37, 18, 28, 38, 48 (impacted tooth); orthodontic treatment; crown lengthening (#12), ridge augmentation in #21 area; implant-supported crown (#46); all-ceramic crowns (#34–44, 14, 13, 23); all-ceramic FPD (#12 P^21^ 22)	The patient showed a pleasing facial appearance after finishing the treatment.
Seymour DW et al, 2012^[37]^-UK	A 48-yr-old woman	Root canal obliteration; FPD: #13P^12^11, failing FPD: #33P^34^P^35^36, crowns: #15, 14, 21–23, 32–43, 46, failing crown: #47; missing teeth #16–18, 26, 27, 37, 38; developmentally absent: #44, 45; residual roots #24, 25, 84, 85	Removed FPD:33P^34^P^35^36, new crown (#33), extracted: #34–36; crown: #47; extracted: #24, 25 (residual roots), 23 (insufficient coronal structure) and 36 (caries); implants (#16, 17, 23–26, 34–36, 44–46)	Not reported
Ayyildiz S et al, 2013^[44]^-Turkey	An 18-yr-old man	Toothwear scale 2–3; SSCs on #16, 26, 36, 46 with secondary caries	SSCs removal and GIC restoration (#16, 26, 36, 46; secondary caries); metal-ceramic crowns (#14–17, 24–27, 34–37, 44–47); all-ceramic crowns (#13–23, 33–43)	At 12 mo, no pathoses associated with rehabilitation.
Bencharit S et al, 2014^[10]^-USA	A 33-yr-old woman	Tooth wear scale 0–3; pulpal obliteration (all the teeth); missing teeth #17, 24, 27, 36, 37, 46, 47; fracture teeth #16, 38, 35, 45; defective restorations: #16, 15; defective FPD: #23P^24^25; residual root #48	Extracted 38 (fracture tooth), 48 (residual root); implant-supported all-ceramic crown (#24); implant-supported metal-ceramic splinted crowns (#36 and 37, 46 and 47); metal-ceramic crowns (#14–16, 25, 26); all-ceramic crowns (#13–23, 34, 35, 44, 45); all-ceramic veneers (#33–43)	Not reported
Campanella V et al, 2018^[31]^-Italy	A 17-yr-old boy	Tooth wear scale 3; partial obliteration of pulp space	Indirect composite resin overlays (#17–14, 24–27, 34–37, 44–47); Empress ceramic veneers (#13–23, 33–43)	At 13 yr, there was a kind of aging but without adhesive failure.
Soliman S et al, 2018^[13]^-Germany	A 10-yr-old girl	Tooth wear scale 3–4; calcified root canals	Composite resin buildups core retained by self-threading parapulpal retentive pins (#12–22, 16, 26, 36, 46); indirect composite restorations (#17–27, 37–47)	At 1.5 yr, secondary caries in #37, 47.
Fan F et al, 2019^[34]^-China	A 20-yr-old woman	Tooth wear scale 3–4: #13-23, #33–43; pulpal obliteration; gingival hyperplasia in the anterior maxillary area	Crown lengthening of #33–43; gingivoplasty (#13–23); all ceramic crowns (#13–23, 33–43)	At 3 mo, no postoperative complications occurred.
Shi S et al, 2020^[30]^-China	A 19-yr-old woman	Tooth wear scale: 0–1; obliterated pulp chambers and root canals	Crown lengthening surgery in maxillary anterior region, maxillary labial frenuloplasty, all-ceramic crowns (#14–24, 34–44)	At 1 yr, a small defect on the edge of the #12, no obvious abnormalities on the restorations.
Milanović M et al, 2021,^[45]^ Serbia	A 22-yr-old woman	Tooth wear scale: 2–3; completely obliterated pulp chambers; a loss of VDO; missing teeth (# 16, 13–21, 31, 35–37, 41, 45, 46)	GIC restorations (# 14, 15, 17, 24–26); extracted: # 23, 27, 47; removable partial dentures;	At 1 yr, the removable partial dentures did not need realignment.
Azpiazu-Flores FX et al, 2022^[15]^-USA	A 20-yr-old man	Tooth wear scale 2–4; obliterated pulp chambers; multiple periapical radiolucent lesions on the maxillary teeth	Maxillary and mandibular extractions (#17–27, 31–35, 37, 41–45, 47); orthognathic surgery with immediate complete denture and acrylic splint, dental implants and implant-supported full-arch prostheses	At 1 yr, no functional limitation or discomfort.

DI = dentinogenesis imperfect, FPDs = fixed partial dentures, GIC = glass ionomer cement, SSCs = stainless steel crowns, VDO = vertical dimension of occlusion.

## Author contributions

**Conceptualization:** Yizhou Zhang, Xiaoting Jin, Baiping Fu.

**Data curation:** Zhengyi Zhang, Sai Hu, Wenxiang Jiang, Haisong Pan.

**Formal analysis:** Zhengyi Zhang, Sai Hu, Wenxiang Jiang, Haisong Pan.

**Supervision:** Ling Zhang, Baiping Fu.

**Writing – original draft:** Yizhou Zhang, Xiaoting Jin.

**Writing – review & editing:** Ling Zhang, Baiping Fu.
